# Egg sanitation with ginger and garlic solutions affects embryonic development, hatchability, blood parameters and post-hatch performance of Japanese quail

**DOI:** 10.1007/s11250-025-04556-8

**Published:** 2025-08-04

**Authors:** M. M. A. El-kashef, Mohamed I. El Sabry

**Affiliations:** 1 Animal and Poultry Production Department, Faculty of Environmental Agricultural Sciences, Arish University, Al-Arish, Egypt; 2 Department of Animal Production, Faculty of Agricultural, Cairo University, Giza, 12613 Egypt

**Keywords:** Disinfectant, Embryonic development, Microbial load, Cholesterol, Hatchability, Post-hatch performance

## Abstract

This study evaluated the effects of hatching egg sanitation with different solutions contain garlic and ginger oils on embryonic development, hatchability, chick quality, and post-hatch performance of quail chicks. Four hundred and fifty quail-hatching eggs were obtained from a 15-week-old flock over 3 consecutive days and stored for a week in a controlled environment at 18 °C and 75% relative humidity for 7 days. Then, hatching eggs were distributed into five groups of 90 eggs. Eggs of the 1st group served as a control group (sanitized with TH4). The eggs of the 2nd and 3rd groups were sprayed with 1 m and 2 ml garlic / L of water, respectively. The eggs of the 4th and 5th groups were sprayed with 1 m and 2 ml ginger oil / L of water, respectively. Eggs were treated within an hour after collection and then stored for a week. Regardless of the treatment and dose, embryos’ weight, hatchability%, and chick quality parameter values of treated groups were significantly higher than those of the control group. Compared to the control group, blood proteins, thyroxin, and total lipids increased in the treated groups (*p* ≤ 0.05). While lower liver enzyme and glucose levels were found in the garlic and ginger sprayed groups. At the 2nd week of age, the growth performance of the treated groups’ chicks, including the body weight, feed intake, body weight gain, and feed conversion ratio surpassed that of the control group chicks. Conclusively, it seems that garlic and ginger solutions are potential natural-produced disinfectants that could reduce the microbial load on the eggshell and improve embryonic development, hatchability, blood constituents, and early growth performance post-hatch. Moreover, using such natural alternatives is an environmental solution that may reduce the hazards of excessive use of chemicals, and could be a viable alternative to chemical disinfectants in quail hatcheries.

## Introduction

Egg and meat production from quail farming is important due to its excellent nutritional value and low-cost investment. Thus, quail farming has been expanded in different countries worldwide (El Sabry et al. 2017; Sabry et al. 2022). Hatchability is a significant index in poultry production. However, several factors affect the embryonic mortality and hatchability percentages as well as the post-hatch performance of chicks, including pathogens, breeder age, hygiene, and incubation conditions (El Sabry et al. 2013; Sabry et al. 2015; Hamouda et al. 2023a).

However, bacterial diseases are among the real challenges that hinder the expansion of poultry production (Mehaisen et al. 2016). The contamination of the hatching egg occurs at the end of the egg tract, due to the contact of eggshell with:1) feces at the end of the oviduct, 2) bedding after oviposition, and 3) during handling of hatching eggs. The presence of many different bacteria *e.g. E.coli* and *Salmonella spp*., on the eggshell’s surface, raises the threat of contamination of the eggs. Bacteria, which penetrate the eggshell finally reduce the hatchability% and quality of chicks. Thus, using an efficient disinfectant in the sanitation process is an important control point to achieve good outcomes (Ibrahim et al. 2018; Hamouda et al. 2023b).

In this regard, different egg sanitizers and routes have been utilized to decrease the microbial load on the eggshell surface e.g. fumigation by formaldehyde or spraying nano-silver (Ibrahim et al. 2018) showed high efficacy. However, the toxic effect of these methods on the chicken embryos and the uncertainty about the safety of nano-materials (El Sabry et al. 2018; Sabry et al. 2021; Issa et al. 2024) encourage the researcher to find natural-origin and eco-friendly disinfectants.

Ginger is one of the potent materials that can be used as an alternative egg sanitizer because it contains anti-microbial, anti-parasitic, and anti-bacterial effects such as Paradol, Zingerone, and Zerumbone (Abdul et al. 2008; Ghazalah and Ali 2008; Kumar et al. 2014), and antiseptic materials (Ali et al. 2008). Moreover, ginger is nutrient-rich such as fatty acids, amino acids, minerals such as iron, calcium, magnesium, selenium, and zinc, and vitamins E and C (Shirin and Jamuna 2010). In addition, ginger contains some biologically active compounds like terpene and phenolic compounds (Stoner 2013; Liu et al. 2019).

Another potential alternative is garlic (*Allium sativum*) extract, which contains anti-bacterial compounds e.g. Allicin and garlicin (Farbman et al. 1993; Reuter et al. 1996; Astal and Younis 2003). In addition, garlic contains several useful compounds such as amino acids and their glycosides, minerals (calcium, phosphorus, potassium, magnesium, sodium, and selenium), and vitamins (A, C, D, E, and B complex vitamins) (Grela and Klebaniuk 2007). Besides, garlic oil showed improved embryonic growth, hatchability%, hatch chick’s weight, and performance of chick (Fouad et al. 2018).

Therefore, this experiment aimed to evaluate the effects of spraying hatching quail eggs with ginger and garlic oil as natural disinfectants on the microbial load on the eggshell. Also, the influence of these natural potential disinfectants on embryonic development, hatchability % blood constituents, and early post-hatch performance were studied.

## Materials and methods

### Solutions Preparation

Different concentrations of garlic and ginger oil solutions were prepared by mixing 1000 ml distilled water with 1 ml or 2 ml garlic or ginger oil (Raw natural oil from Herbo-Ridouane laboratoire). To ensure that the oil is distributed in the water in a homogeneous and stable manner. where, Essential oil and emulsifier (Tween 80) were mixed by magnetic stirrer for 5 min at 500 rpm and the distilled water containing 0.8% citric acid was added slowly to the mixed oil during mixing, with increase the number of turns to 700 rpm for 15 min (McClements 2005).

### Egg collection

Eggs were collected for 3 consecutive days. The eggs were sanitized, within 1 h of egg collection, with different concentrations of garlic or ginger solutions. After sanitization, eggs were stored in a controlled environment at a temperature of 18 °C and humidity levels of 75% for 7 days.

### Egg treatments

Four hundred and fifty hatching Japanese quail eggs (*Coturnix Japonica*) were obtained from a 15 weeks-old of age flock. Eggs were randomly divided into five groups after stored 7 days to; 90 eggs each group, 3 replicates 30 eggs each. Eggs in the 1st group were sprayed with TH4 and served as a control group. The 2nd and 3rd groups were sprayed with 1 ml/L and 2 ml/L of garlic solutions, respectively. The 4th and 5th groups were sprayed with 1 ml/l and 2 ml/L ginger solutions, respectively. The eggs were allowed to dry at 22 °C for 20 min. Eggs were incubated under standard conditions by using hatching machines PTO-C8 (Egyptian manufacturer), automatically turned every 2 h, during the first 14 days of the incubation period, the temperature was 37.5˚C and RH ranged between 55 and 60% in the setter, while temperature was 36.5 ˚C and 70–80% RH in the hatcher.

### Microbiological examination

After spraying eggs, they were allowed to dry at 22 °C for 20 min. Then, five eggs per group were taken for bacteriological examination at 7 and 14 days of incubation. Each egg was placed immediately in a sterile bag containing 10 ml of sterile phosphate-buffered saline (PBS) (pH 7.2). This whole-egg washing technique was applied to recover the shell-associated bacteria for estimating the total viable bacterial count (TBC) by using plate counting agar (PCA) (Conda lab., Spain). Serial dilutions were made in PBS and then were cultivated into sterile petri plates (Jones et al. 2002). Then, plates were incubated at 37 °C for 24 h. Bacterial colonies were counted and multiplied by the dilution factor. Colonies were measured as cfu/egg (Hamouda et al. 2023b).

$$\begin{aligned}&\:\mathbf{E}\mathbf{g}\mathbf{g}\:\mathbf{w}\mathbf{e}\mathbf{i}\mathbf{g}\mathbf{h}\mathbf{t}\:\mathbf{l}\mathbf{o}\mathbf{s}\mathbf{s}\:\varvec{\%}\cr&\quad=\frac{\begin{aligned}&\text{I}\text{n}\text{i}\text{t}\text{i}\text{a}\text{l}\:\text{e}\text{g}\text{g}\:\text{w}\text{e}\text{i}\text{g}\text{h}\text{t}\cr&\quad-\text{e}\text{g}\text{g}\:\text{w}\text{e}\text{i}\text{g}\text{h}\text{e}\text{d}\:\text{a}\text{t}\:\text{d}\text{a}\text{y}\:14\:\text{o}\text{f}\:\text{i}\text{n}\text{c}\text{u}\text{b}\text{a}\text{t}\text{i}\text{o}\text{n}\end{aligned}}{\text{I}\text{n}\text{i}\text{t}\text{i}\text{a}\text{l}\:\text{e}\text{g}\text{g}\:\text{w}\text{e}\text{i}\text{g}\text{h}\text{t}}\times\:100\end{aligned}$$

### Embryonic development and mortality %

For embryonic development determination, 5 eggs from each group were taken randomly at day 14 of incubation period. The relative of embryo’s weight were calculated = embryo weight/ egg weight*100. Also, embryonic length, and embryonic shank length were recorded.

At hatch, all chicks were removed and weighed. Un-hatched eggs were opened to determine embryonic mortality %. The hatchability % of fertile eggs was calculated.

$$\:\text{E}\text{m}\text{b}\text{r}\text{y}\text{o}\text{n}\text{i}\text{c}\:\text{m}\text{o}\text{r}\text{t}\text{a}\text{l}\text{i}\text{t}\text{y}\left(\%\right)=\frac{\text{N}\text{u}\text{m}\text{b}\text{e}\text{r}\:\text{o}\text{f}\:\text{d}\text{e}\text{a}\text{d}\:\text{e}\text{m}\text{b}\text{r}\text{y}\text{o}\text{s}\:}{\text{N}\text{u}\text{m}\text{b}\text{e}\text{r}\:\text{o}\text{f}\:\text{f}\text{e}\text{r}\text{t}\text{i}\text{l}\text{i}\text{z}\text{e}\text{d}\:\text{e}\text{g}\text{g}\text{s}\:}\times\:100$$

$$\:\text{H}\text{a}\text{t}\text{c}\text{h}\text{a}\text{b}\text{i}\text{l}\text{i}\text{t}\text{y}\:\text{o}\text{f}\:\text{f}\text{e}\text{r}\text{t}\text{i}\text{l}\text{e}\:\text{e}\text{g}\text{g}\text{s}\left(\%\right)=\frac{\text{N}\text{u}\text{m}\text{b}\text{e}\text{r}\:\text{o}\text{f}\:\text{h}\text{a}\text{t}\text{c}\text{h}\text{e}\text{d}\:\text{c}\text{h}\text{i}\text{c}\text{k}\text{s}\:}{\text{N}\text{u}\text{m}\text{b}\text{e}\text{r}\:\text{o}\text{f}\:\text{f}\text{e}\text{r}\text{t}\text{i}\text{l}\text{i}\text{z}\text{e}\text{d}\:\text{e}\text{g}\text{g}\text{s}\:\text{s}\text{e}\text{t}}\times\:100$$

### Physiological parameters

On hatching day, six chicks per group were randomly selected and sacrificed. The internal organs, including the residual yolk sac, liver, gizzard, heart, and intestine, were dissected and weighed. Then, the relative organs’ weight was expressed as a percentage of the live body of chicks. Also, blood samples were obtained from jugular vein. Serum was obtained from the blood samples by centrifugation for 20 min at 3500 rpm and stored at − 20 Cº until used in the analysis. Blood biochemical parameters of each parameter were measured according to the kit manufacturers’ guidelines. Total protein, albumin, total lipids, cholesterol, and glucose concentration in blood serum were measured according to the methods described by Sonnenwirth and Jarett (1980), Doumas (1971), and Stein (1986), respectively.

### Post –hatch performance of chicks

Thirty chicks were grown for 14 days post-hatch. Chicks were weighed and raised in separate groups. During this period, birds received diets containing 24% CP and 2900 Kcal ME/ Kg (NRC 1994). Feed and fresh water were provided ad-libitum. The temperature was set at 33 °C and the lighting period was 23 h light/ 1 h dark. Feed intake (FI) was recorded for each group. As well as chicks were weighed individually at d 1 and 14 of age. Finally, the feed conversion ratio (FCR) was calculated.

### Statistical analysis

One-way ANOVA was used to statistically evaluate the collected data by following the General Linear Model (GLM) Procedure outlined in the SAS User’s Guide, using the following model, Yij = µ + Ti + eij. Where: Yij: The j^th^ observation of the i^th^ treatment. µ: The overall mean. T_i_: The effect of the i^th^ treatment. E_ij_: Random error. Duncan’s multiple range test was used to examine significance levels for mean differences at (*P* ≤ 0.05) (Duncan 1955).

## Results and discussion

### Bacterial count

Hatching eggs with a minimum microbial load is key for the succeeding hatching process. A variation in the contamination level of hatching eggs, from 10^2^ to 10^7^, can be observed. Moreover, contaminated eggshells by harmful bacteria could be a source of spreading diseases and result in high embryonic and chick mortalities and low hatchability %. In the current study, the total bacterial count on the eggshell that was disinfected with either garlic or ginger solution was significantly lower compared to that of the eggs of the control group (Table 1). Moreover, using higher concentrations of 2 ml/L of garlic and ginger solution significantly reduced the microbial count on the eggshell compared to using 1 ml/L of garlic or ginger solution. These results could be due to the effective antimicrobial substances such as Paradol, Zingerone, and Zerumbone (Abdul et al. 2008), which may lead to bacteria deactivation by reactions with the bacteria cell wall, and protein denaturation. Also, Iwalokun et al. (2004) and Gbenga et al. (2009) reported that antimicrobial compounds in garlic oil could reduce the load of harmful bacteria on the eggshell that affect the livability of embryos % and negatively affect the hatchability rate (Baylan et al. 2018).

**Table 1:** Effect of spraying Japanese quail eggs with garlic and ginger solution on the total bacterial counts (X 10^3^ Cfu /egg) after 7and 14 days of incubation period

Time	Treatment					
	Control	Garlic solution		Ginger solution		Sig.
		1 ml/L	2 ml/L	1 ml/L	2 ml/L	
At d 7	3.6 ± 2.2^a^	2.1 ± 2.1^b^	1.6 ± 1.5^c^	2.1 ± 2.8 ^b^	1.5 ± 2.0 ^c^	*
At d 14	3.8 ± 4.3 ^a^	2.2 ± 3.1 ^b^	1.5 ± 1.5^c^	2.0 ± 2.3 ^b^	1.4 ± 1.7 ^c^	*

^a, b,c^ Means ± SE in the same row with different superscripts are significantly different (*p* ≤ 0.05)

NS = Not significant, * (*P* ≤ 0.5) significant, ** (*P* ≤ 0.1) highly significant

### Embryonic development and hatching parameters

Results in Table 2 showed that eggs sprayed with either garlic or ginger solution had significantly less egg weight loss (≥ 1%) compared to control eggs. Similar results were reported by Fouad et al. (2018) and Abo-Samaha and Basha (2021). Covering the egg pores with garlic oil reduces the evaporation of water from eggs during the incubation period (Shahein and Sedeek 2014). This can be a solution for maintaining the moisture of eggs, especially those of quail with thin eggshells.

**Table 2:** Effects of spraying quail eggs with garlic and ginger solutions on embryonic development at day 14 of incubation period and hatchability %

Traits	Control	Garlic solution		Ginger solution		Sig.
		1 ml/L	2 ml/L	1 ml/L	2 ml/L	
Egg weight loss (%)	11.91 ± 1.17^a^	9.72 ± 0.9 ^b^	9.61 ± 0.9 ^b^	9.76 ± 0.7 ^b^	9.59 ± 1.0 ^b^	*
Relative embryo w. (%)	36.11 ± 1.37^c^	41.8 ± 1.2^ab^	43.2 ± 1.76 ^a^	40.4 ± 1.3 ^b^	42.0 ± 1.3 ^a^	*
Embryo length (cm)	6.5 ± 0.35^c^	6.9 ± 0.4 ^ab^	7.2 ± 0.3 ^a^	6.9 ± 0.4 ^ab^	7.0 ab ± 0.4^ab^	*
Shank length (cm)	0.98 ± 0.01^c^	1.22 ± 0.0^ab^	1.35 ± 0.1 ^a^	1.18 ± 0.0 ^ab^	1.31 ± 0.0^ab^	*
Hatchability of fertile (%)	80.6 ± 2.33 ^b^	88.8 ± 3.1^a^	90.4a ± 1.3 ^a^	88.5 ± 2.3^a^	90.7a ± 3.2 ^a^	*
Embryonic mortality (%)	17.9 ± 1.53 ^a^	11.5 ± 1.2 ^bc^	8. 7d ± 1.4	13.1 ± 1.3 ^b^	9.8 ± 1.1^cd^	*

^a, b,c^ Means ± SE in the same row with different superscripts are significantly different(*p* ≤ 0.05)

NS = Not significant; ‎* (*P* ≤ 0.5) significant, ** (*P* ≤ 0.1) highly significant

Generally, spraying eggs with either garlic or ginger solution significantly improved embryos’ weight% and length and shank length compared to those of embryos in control. Moreover, the embryo weight% (40.4 ± 1.3) of the 1 ml ginger /L was the lowest among the treatments. These results agree with the findings of Fouad et al. (2018) and Abo-Samaha and Basha (2021), who found lower weight loss % and improvement in the embryos’ weight and length and shank length and hatchability% in quail due to sanitizing quail eggs using garlic oil. Moreover, they reported that the values of egg weight loss %, hatch time, and embryonic mortality % of the garlic oil-treated group were lower than those of control eggs.

High embryo weight% in the treated groups could be correlated with lower egg weight loss during the incubation period. Similarly, Peebles et al. (1987) and Davis et al. (1988) indicated that increasing the egg weight loss of incubated eggs reduces the weight of chicks due to less available water in eggs, which is absorbed in new tissue and increases the chick’s weight.

The hatchability of fertile eggs %, of all garlic and ginger groups was significantly higher than that of the control group. In addition, it seems that using a higher dosage of either garlic or ginger oil was more efficient. Similarly, Fouad et al. (2018) and Abo-Samaha and Basha (2021) found an improvement in the hatchability% of quail eggs due to sanitizing eggs with garlic oil. In chickens, an improvement in hatchability % was reported by Rizk et al. 2022, when they sprayed with 2 and 3 ml garlic oil /L solutions. In this context, Baylan et al. (2018) indicated that the immersion of eggs in garlic extract at rates of 2.5% and 5.0% may be used as an alternative to formaldehyde fumigation for the disinfection of hatching quail eggs, without enhancing hatching performance. In addition, using ginger solution (ginger oil 5%) for egg disinfection lowered embryonic mortality % and improved hatchability % (Al-Shammari et al. 2022). It could be suggested that anti-microbial, anti-parasitic, and anti-bacterial (Ghazalah and Ali 2008; Kumar et al. 2014), and antiseptic compounds (Ali et al. 2008) in ginger solution may lessen the microbial load on the eggshell and enhance the immunity of embryos.

At the same time, lower embryonic mortality may refer to the beneficial compounds in the garlic and ginger oils that may support the embryo’s development and enhance its livability. In this regard, Mahjar and Al-Salhie (2022) showed that embryonic mortality % was significantly decreased due to in-ovo injection with alcoholic garlic extract 50, 100, and 150 µl/ egg than this of the control group. This decline in embryonic mortality and improvement in hatchability % may show a prevalence of many active organic sulfur compounds, which may act as antioxidants. These compounds may alleviate the stressors during embryonic life and improve the health status of embryos (Fanelli et al. 1998; Siegers et al. 1999; Fadlalla et al. 2010). The abovementioned results may prove the profitability and safety of using garlic oil in sanitizing hatching eggs.

### Chick quality and organs weight at hatch

Regardless of the type of solution and dose of treatments, results in Table 3 showed that chick weight, chick shank length, chick body length, and residual yolk sac % results of the treated groups were better than those of the control group. These results agree with the findings of Fouad et al. (2018), Abo-Samaha and Basha (2021), Al-Shammari et al. (2022), and Rizk et al. (2022), who used different doses of garlic and ginger solution in hatching egg sanitation. Moreover, residual yolk weight% was significantly lower in treated groups compared to control groups. El-Kashef (2021) and El-kashef (2022a, b) reported that the use of ginger in quail diets led to a significant improvement in the immunity, growth performance, and egg production. Regardless of the type of solution and dose, higher chick weights in treated groups could be due to the lower egg weight loss % and higher embryo weight in the treated groups.

Also, better chick quality parameters and lower yolk weigh % could be due to the entry of garlic and ginger solutions through the pours of eggshells. These solutions might enhance the embryo to utilize the yolk and work as a growth prompter as it contains several beneficial compounds. For instance, garlic contains beneficial compounds such as amino acids and their glycosides, calcium, phosphorus, potassium, magnesium, sodium, selenium, and A, C, D, E, and B complex vitamins. Besides, garlic contains anti-bacterial, antioxidant, anti-inflammatory, and cardiovascular-protective compounds as proven by several studies. Similarly, ginger contains rich nutrients such as fatty acids, amino acids, and minerals e.g. Fe, Ca, Mg, Se, and Zn, and vitamins E and C (Shirin and Jamuna 2010). In addition, ginger exhibited many bioactive compounds like terpene and phenolic compounds, which have antioxidant effects (Nile and Park 2015; Liu et al. 2019).

**Table 3:** The effect of spraying quail hatching eggs with Garlic and ginger solutions on chick quality parameters and relative weight of organs %

Traits	Control	Garlic solution		Ginger solution		Sig.
		1 ml/L	2 ml/L	1 ml/L	2 ml/L	
Chick weight (g)	9.2 ± 1.1 ^c^	9.4 ± 1.1^b^	9.6 a ± 1.2^a^	9.4 ± 1.0^b^	9.5 ± 1.1^ab^	*
Chick length (cm)	9.8 ± 0.6 ^c^	10.8 ± 0.6^b^	11.2a ± 0.6 ^a^	10.8 ± 0.6^b^	11.1 ± 0.6^a^	*
Shank length (cm)	1.8 ± 0.3^c^	2.1 ± 0.2^ab^	2.2 ± 0.2^a^	2.0 ± 0.2 ^b^	2.2 ± 0.2^a^	*
Yolk residual %	8.29 ± 0.33^a^	7.21 ± 0.18 ^b^	6.38 ± 0.19 ^c^	7.54b ± 0.36 ^b^	6.74 ± 0.22^c^	*
Intestine %	2.27 ± 0.2^c^	2.68 ± 0.1^a^	2.77 ± 0.2^a^	2.53b ± 0.1 ^b^	2.69 ± 0.2^ab^	*
Liver %	2.45 ± 0.18 ^b^	2.63 ± 0.12^a^	2.71 ± 0.21^a^	2.65 ± 0.11^a^	2.69 ± 0.24^a^	*
Heart %	0.69 ± 0.05^b^	0.87 ± 0.03^a^	0.91 ± 0.05^a^	0.82 ± 0.02^a^	0.88 ± 0.06^a^	*
Gizzard %	4.62 ± 0.51	4.61 ± 0.43	4.65 ± 0.57	4.60 ± 0.42	4.65 ± 0.52	**NS**

^a, b,c^ Means ± SE in the same row with different superscripts are significantly different (*p* ≤ 0.05)

NS = Not significant; ‎* (*P* ≤ 0.5) significant, ** (*P* ≤ 0.1) highly significant

At hatch, the relative yolk sac weight of chicks of the control group was the highest compared to this of chicks from the treated groups. This result agrees with the findings of Rizk et al. (2022), who sprayed hatching with garlic extract 3 ml garlic/ L.

Relative weights of the intestine, liver, and heart of chicks from treated groups were higher compared to those of chicks from the control group, while the gizzard weight% was not influenced by treatments. These results agree with the finding of Fouad et al. (2018), who reported that spraying hatching eggs with garlic oil significantly increased the relative weight of the intestine, liver, and heart. Whereas, Rizk et al. (2022) reported that spraying hatching eggs with garlic solution (3 ml/litter) did influence liver heart and gizzard weights %. However, the treatment significantly reduced yolk residual and bursa weight% compared to other groups.

These results may be due to garlic containing the main components like organic sulfurous compounds. Additionally, allicin is a strong antibiotic and it is effective against bacteria both of them negative and positive. Also, Garlic contains several useful compounds such as amino acids and their glycosides, calcium, phosphorus, potassium, magnesium, sodium, selenium, and A, C, D, E, and B complex vitamins. Plus, Garlic has anti-bacterial (Iwalokun et al. 2004; Gbenga et al. 2009), anti-stress agent (Baylan et al. 2018), anti-oxidant (Fanelli et al. 1998; Siegers et al. 1999), anti-cancer, anti-inflammatory, and cardiovascular-protective properties (Reuter et al. 1996). These components play an important role in reducing the microbial load on the eggshell which leads to the improved health status of embryos as reported by (Fadlalla et al. 2010). It is also believed that the increase in the internal organs percentage for the hatched chicks is due to the increase in embryo weight at hatching, as a result of the role played by the components of the garlic in maintaining the eggs losing ratio during the incubation period, where the studies indicated that increasing the weight loss of incubated eggs reduces the weight of chicks due to water evaporation from eggs (Peebles et al. 1987; Davis et al. 1988).

On the other hand, the improvement resulting from the use of the ginger solution by spraying on quail fertile eggs in carcass constituents may be due to ginger containing rich nutrients such as fatty acids, amino acids, minerals such as iron, calcium, magnesium, selenium, and Zinc, and important vitamins such as vitamins E and C (Shirin and Jamuna 2010). in addition, ginger contains some biologically active compounds like terpene and phenolic compounds (Liu et al. 2019), Also, ginger exhibits many bioactivities like anti-microbial, anti-parasitic, and anti-bacterial effects (Ghazalah and Ali 2008; Kumar et al. 2014), and antiseptic materials (Ali et al. 2008). These components perform the same role that garlic solution played in reducing the microbial load on the eggshell and increasing in embryo’s weight at hatching. This may lead to an increase in the internal organs of hatched chicks. Additionally, this improvement may be due to the anti-oxidant activities of some components of ginger (Rocha et al. 2010) and to the capacity of ginger contents to modulate the immune system (Salem 2005).

### Blood biochemical parameters

Results in Table 4 showed that values of total protein, albumin, globulin, and HDL-cholesterol for hatched chicks of treated groups were higher than those of the control group. While, total cholesterol, LDL- cholesterol, glucose, ALT, and AST were significantly decreased for all treated groups compared to the control. Also, the thyroxine hormone recorded a significant increase for treated groups with ginger and garlic compared to the control groups.

**Table 4:** The effect of spraying hatching eggs with garlic and ginger solution on the blood biochemical parameters of quail chicks

Blood parameters	Control	Garlic solution		Ginger solution		Sig.
		1 ml/L	2 ml/L	1 ml/L	2 ml/L	
T. protein (g/dl)	3.1 ± 0.3^b^	3.5 ± 0.2 ^a^	3.6 ± 0.3^a^	3.5 ± 0.2^a^	3.6 ± 0.3 ^a^	*
Albumin (g/dl)	1.63 ± 0.15 ^b^	1.75 ± 0.03 ^a^	1.82 ± 0.16 ^a^	1.83 ± 0.05 ^a^	1.89 ± 0.1 ^a^	*
Globulin (g/dl)	1.48 ± 0.26^b^	1.76 ± 0.37 ^a^	1.81 ± 0.24 ^a^	1.69a ± 0.34	1.7 ± 0.2 ^a^	*
Glucose (mg/dl)	220 ± 5 ^a^	195 ± 4 ^b^	185 ± 3 ^bc^	193 ± 2 ^b^	181 ± 3^c^	*
Total cholesterol (mg/dl)	142.5 ± 4.1 ^a^	143 ± 3 ^a^	142 ± 4 ^a^	135 ± 4^b^	128 ± 2 ^c^	*
HDL-cholesterol (mg/dl)	82.0b ± 4.3^b^	92.3 ± 2.5 ^a^	93.8 ± 3.7 ^a^	94.9 ± 4.36 ^a^	96.4 ± 4.6 ^a^	*
LDL-cholesterol (mg/dl)	60.4 ± 4.3 ^a^	51.3 ± 4.7 ^b^	49.1 ± 3.5^b^	40.2 ± 4.0 ^c^	32.3 ± 3.7^d^	*
ALT (U/L)	15.1 ± 2.1 ^a^	12.5 ± 2.2^d^	11.7 ± 1.2^e^	12.7 ± 1.1 ^c^	11.6 ± 1.10 ^e^	*
AST (U/L)	22.8 ± 2.5 ^a^	18.5b ± 1.6^b^	16.8 ± 1.50 ^cd^	17.9 ± 2.61^bc^	16.2 ± 1.25^d^	*
Thyroxin (ng/ml)	9.3 ± 0.3 ^b^	10.2 ± 0.2 ^ab^	11.3 ± 0.4 ^a^	10.1 ± 0.5 ^ab^	10.8 ± 0.6 ^a^	*

^a, b,c^ Means ± SE in the same row with different superscripts are significantly different(*p* ≤ 0.05)

NS = Not significant; ‎* (*P* ≤ 0.5) significant, ** (*P* ≤ 0.1) highly significant

The findings align with those of Fouad et al. (2018), who reported a significant improvement in blood biochemical constituents when garlic oil solutions were sprayed on fertile quail eggs. Similarly, Rizk et al. (2022) observed a notable enhancement in blood biochemical constituents specifically in the group of eggs treated with a garlic solution at a concentration of 3 ml/liter, compared to the other groups.

Spraying garlic oil on fertile eggs resulted in the highest rate of beneficial blood components in hatched chicks. This improvement may be attributed to the optimal low-density lipids and low-density cholesterol levels (Fadlalla et al. 2010). Additionally, garlic has been found to lower blood cholesterol levels in poultry (Khan et al. 2012). It is suggested that the active organic sulfur compounds in garlic may possess antioxidant properties (Fanelli et al. 1998; Siegers et al. 1999) and enhance liver function, as indicated by a reduced profile of liver enzymes profile. Also, garlic serves as a cardiovascular-protective agent (Reuter et al. 1996). The observed improvement in blood biochemical components could also be linked to the overall health of the embryos, which likely benefited from the use of a garlic oil solution sprayed on fertile eggs, as reported by Fadala et al. (2010).

The improvement in blood biochemistry may be attributed to the presence of minerals, vitamins, and biologically active components in ginger, in addition to its roles as an antioxidant, anti-inflammatory agent, antimicrobial substance, and protector of cardiovascular health. The increase in total proteins could be due to the phenolic compounds found in ginger, such as gingerol, gingerdione, and shogaols, which enhance immune responses through their potent antioxidant properties. Furthermore, the rise in the level of blood globulin, an indicator of immune response and a source of antibodies (Abdel Fattah et al., 2008), may be linked to the production of immunoglobulins. Therefore, the observed effects could be due to high immunoglobulin concentration (Habibi and Ghahtan 2019).

The decrease in plasma cholesterol levels may be attributed to ginger showing a strong anti-lipidemic effect on triglyceride levels and cholesterol (Jang et al. 2007); hence, its mode of action may be related to the inhibition of cholesterol synthesis such as hydroxy-methylglutaryl coenzyme A (Saeid et al. 2010), to cause liver-specific inhibition of cholesterol synthesis (Manju et al. 2006).

Furthermore, the reduction in glucose levels may be attributed to the anti-diabetic effects of ginger. Research suggests that these properties are linked to the activation of adenosine monophosphate kinase (Haddad et al. 2003; Sanz 2008). Moreover, when the liver is injured, it releases the enzymes ALT and AST into the bloodstream (Kaplan et al. 2003). Therefore, given ginger’s anti-inflammatory effects, it may influence liver enzyme levels and contribute to liver health.

### Post-hatch performance of chicks

Spraying quail eggs with garlic and ginger solutions significantly improved the FI, BW, BWG, and FCR of quail chicks compared to those of chicks in the control group at 2 wk of age (Table 5). These results could be due to the penetration of the solution and mixing with internal egg content. Similar findings were reported by Baylan et al. (2018), Fouad et al. (2018) and Rizk et al. (2022) found that spraying or immersion of quail eggs in garlic extract at (2.5% and 5.0%) improved final BW and BWG, while FI and FCR were not affected (Baylan et al. 2018). Also, Kumar et al. (2014), Nile and park (2015), Liu et al. (2019) suggested that the positive effect of spraying ginger extract on the post-hatch performances of chicks could be due to its content of active compounds e.g. phytochemicals, nutrients, and antimicrobial, and anti-oxidant compounds. In addition, ginger contains camphon, flavonoids, gingerdiol, gingerol, gingeron, humolin, limonene, and shogaols, which stimulate the digestive enzymes and immunity (Hashimoto et al. 2002; Ghazalah and Ali 2008; El-kashef 2022a, b).

**Table 5:** Shows the effect of spraying quail fertile eggs with garlic and ginger solutions on growth performance for hatched quail chicks at 2 weeks of age

	Control	Garlic solution		Ginger solution		Sig.
		1 ml/L	2 ml/L	1 ml/L	2 ml/L	
BW at d 1 (g)	9.2 ± 1.08^c^	9.4 ± 1.1^b^	9.6 ± 1.2^a^	9.4 ±1.0^b^	9.5 ± 1.1 ^ab^	*
BW at d 14 (g)	65.7 ± 2.2^c^	72.18 ± 2.24^b^	77.12 ± 4.02^a^	71.75 ± 2.14^b^	77.53 ± 3.19^a^	*
FI (g)	75.7 ± 5.3^ab^	76.2 ± 6.9 ^ab^	77.87 ± 5.8^a^	75.8 ± 4.20^ab^	76.4 ± 4.5^ab^	*
BWG (g)	56.6 ± 3.8^c^	63.00 ± 3.3^b^	68.0 ± 6.00^a^	62.58 ± 2.10^b^	68.4 ± 3.2^a^	*
FRC	1.34 ± 0.03^a^	1.21 ± 0.04^b^	1.15 ± 0.03^bc^	1.21 ± 0.01^b^	1.12 ± 0.04^c^	*

^a, b,c^ Means ± SE in the same row with different superscripts are significantly different (*p* ≤ 0.05)

NS = Not significant; ‎* (*P* ≤ 0.5) significant; ** (*P* ≤ 0.1) highly significant

## Conclusion

Natural herbal products seem to be potential natural alternatives for chemical disinfectants of hatching eggs. In this study, the use of studied these products showed no deleterious effect on the hatchability %, chick quality, and early post-hatch performance of chicks. In addition, natural-origin disinfectants may benefit the health of birds and reduce environmental pollution by chemical compounds in the agriculture sector.

## Data Availability

Upon request.
